# Gait Training with Bilateral Rhythmic Auditory Stimulation in Stroke Patients: A Randomized Controlled Trial

**DOI:** 10.3390/brainsci8090164

**Published:** 2018-08-31

**Authors:** Soonhyun Lee, Kyeongjin Lee, Changho Song

**Affiliations:** 1 Department of Physical Therapy, College of Health Science, Sahmyook University, Seoul 01795, Korea; hotpag@hanmail.net; 2 Department of Physical Therapy, College of Health Science, Kyungdong University, Gangwon-do 24764, Korea; kjlee@kduniv.ac.kr

**Keywords:** stroke, rhythmic auditory stimulation, symmetry, gait

## Abstract

The aim of this study was to investigate the effect of gait training with bilateral rhythmic auditory stimulation (RAS) on lower extremity rehabilitation in stroke patients. Forty-four participants (<6 months after stroke) were randomly allocated to the gait training with bilateral rhythmic auditory stimulation (GTBR) group (*n* = 23) and the control group (*n* = 21). The GTBR group had gait training with bilateral RAS for 30 min a day, 5 days a week, in a 6-week period, in addition to conventional therapy. The control group had gait training without RAS, and conventional therapy. Outcome measures included gait symmetry, gait ability, balance ability, and lower extremity function. Gait symmetry on step time showed significant improvements compared to baseline (*p* < 0.05) in the GTBR group, but not in the control group. Gait ability was significantly improved in both groups relative to baseline values (*p* < 0.05), and the GTBR group showed significantly greater improvement in comparison to the control group (*p* < 0.05). Both groups showed significant improvements in the Timed Up and Go test (TUG), Berg Balance Scale (BBS), and Fugl–Meyer Assessment (FMA) compared to baseline (*p* < 0.05). GTBR is an effective therapeutic method of improving symmetric gait in stroke rehabilitation. Moreover, we found that GTBR beat frequency matching fast step time might be even more beneficial in improving gait symmetry. Future studies may develop a method of applying RAS on step time and length for improvement of gait symmetry in stroke patients.

## 1. Introduction

Stroke patients have gait disorders, impairments in the lower extremities, changes in gait pattern, weakness, loss of sensation, spasticity, abnormal movement timing, and balance disorders [1]. Patients with stroke highlight that changes in gait are the most urgent impairment which needs attention. For an effective rehabilitation program, improvement in gait pattern should be included as a primary treatment goal [2]. Moreover, restoration of gait can be viewed as the most significant goal for the rehabilitation of stroke patients [3].

Gait disorders in stroke patients result from several factors, such as declining paretic muscular strength, spasticity, and paralysis [4]. Gait dysfunction in stroke patients can also include limitations with respect to walking speed and movement, due to flexor synergy of the paretic upper extremity as well as weakness of the extensor [5]. Moreover, because of abnormal muscular activity and spasticity, non-paretic and paretic asymmetries appear between separated gait patterns [6]. Additionally, stroke patients show asymmetric gait, because their paretic side moves slower than their non-paretic side in terms of step time. In addition, their paretic side produces a narrower step length compared to the non-paretic side [7]. Due to such asymmetry on their gait, patients may have difficulties with response behaviors to prevent falls when their body is perturbed [6]. If the abnormal gait pattern and asymmetric gait improve, stroke patients might be able to return to previous levels of gait function [4].

With the purpose of improving patients’ gait, numerous studies have used the following themes: Modification of weakened dorsiflexors [8,9], gait training with supported body weight [10], gait training with rhythmic auditory stimulation (RAS) [6,11], gait training utilizing virtual reality [12], gait training using a robot [13], and circuit class therapy [14].

Previous studies have indicated that it is better to increase step length than to produce higher step frequencies for a more efficient gait pattern. The velocity increase of the control group relying more on higher step rates may have been due to the fact that improvements in stride length were compromised in this group by the persistence of high asymmetry in step patterns [15]. RAS may act on more central facilitation mechanisms because the symmetry of stride times and stride length have been shown to improve with RAS [16].

In particular, recent studies applying RAS aimed at improving gait asymmetry (GA) and ability in various ways. RAS indicates an activity that stimulates rhythmic sensation through auditory stimulation, affecting the motor system. This aims to activate each part of the brain by synchronizing motor and sensory areas within a certain duration of stable time [17]. RAS is based on the activity of leading a stable synchronization between rhythmic auditory signals and motor activity with the use of rhythmic signals toward the motor system [15]. Moreover, RAS is a method of gait rehabilitation to facilitate training of rhythmical movement with the use of external rhythmic cues [18].

Studies on RAS have been performed on Parkinson’s disease and stroke patients, mainly utilizing music and metronomes. Thaut et al. [15] conducted a study on stroke patients which concerned gait training using a metronome. This study showed positive results with increased velocity, stride length and cadence in the RAS group. Various studies were subsequently performed with RAS. Schauer and Mauritz [19] performed musical motor feedback (MMF) gait training on hemiplegic patients to assert that RAS was effective on stride length, walking speed, and gait symmetry. In many studies, most of the applied rhythmic cues used a metronome and music, and the application methods were varied [11,20]. It was determined that RAS was effective on gait velocity, stride length, and so on [11,20,21].

Gait training with the beat of the metronome was effective on velocity and stride length. An increase in gait symmetry, which was caused by rhythm application on both legs, was effective in enhancing temporal symmetry [20]. Schauer and Mauritz [19] applied MMF on gait training in stroke patients.

The studies using a metronome and music provided rhythm after producing cadence, and subsequently performed gait training. Thus, the studies could explore coordination between gait ability and auditory stimulation. In recent studies, the application method to RAS was studied and RAS was applied to heel strike during gait cycle.

Gait training in synchronized RAS with heel strikes improved gait ability [22]. Furthermore, in a previous study of heel contact with a metronome beat on a treadmill, greater consistency of leg movement and auditory cues, and at the same time, increased gait symmetry were observed in the group receiving bilateral RAS than in the group receiving unilateral RAS [17].

Additionally, Roerdink et al. [23] made a sudden change to the tempo during gait training on the treadmill, while synchronizing rhythm signals of another tempo with heel strikes of the paretic and non-paretic sides. Here, they found a stability restoration of trunk movement as well as an improvement in gait ability. Although the RAS used in previous studies had different levels of gait velocity and stride length on the paretic and non-paretic sides, it was applied according to an average cadence. The previous studies were performed in a limited space called a “treadmill” [22,23] and utilized various application methods of RAS [11,17].

RAS is designed to give rhythm to prevent a gait while walking in patients with Parkinson’s disease. Previous studies have attempted to identify changes in gait by applying these principles to stroke patients, but the results are unclear. This is because stroke patients do not experience a rhythm problem in walking, like patients with Parkinson’s disease, but an asymmetrical problem. Therefore, in order to solve this problem, this study aimed to measure the step time of the individual without using the metronome applied to the existing studies, to provide the RAS to stroke patients, and to improve the asymmetry of walking. The purpose of this study was to investigate the effects of gait training with bilateral RAS on gait symmetry, walking ability, and balance ability of stroke patients.

## 2. Materials and Methods

### 2.1. Subjects

Participants were inpatients who had developed a stroke at least 6 months previously, and were selected from U rehabilitation center (Gyeonggi Province, South Korea). Individuals were included when they (1) were able to independently walk for a minimum of 10 min, (2) had a Korean version of mini-mental state examination score of over 21, and (3) were able to follow instructions; and excluded when they had (1) auditory system problems and (2) other conditions, such as fractures or digital neuropathy, on the lower extremities. All participants signed informed consent forms after receiving detailed explanations of the study objectives and requirements. The study was approved by the Institutional Review Board of Sahmyook University (date of approval: 12 October 2012; No. SYUIRB2012-059).

### 2.2. Procedure

**Figure 1:**
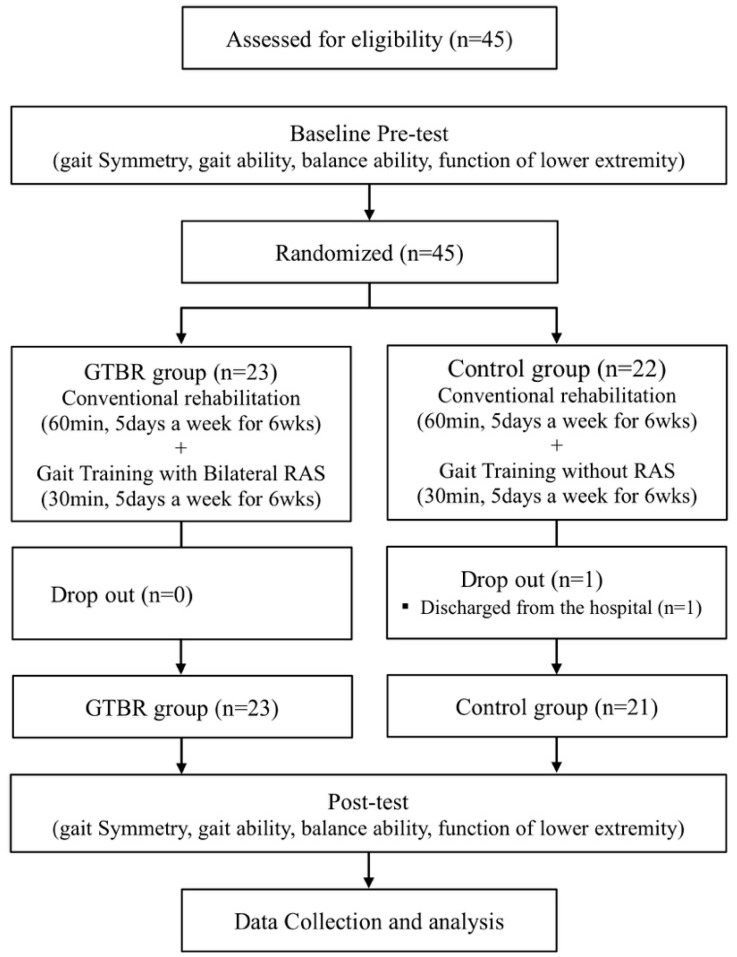
Flow diagram of the study.

A total of 45 participants were enrolled in the trial and randomly assigned to the gait training with bilateral rhythmic auditory stimulation (GTBR) group (*n* = 23) or the control group (*n* = 22) after undergoing a preliminary test. Random Allocation Software was used to minimize selection bias. The GTBR group underwent GTBR for 30 min a day, 5 days a week, for 6 weeks, in addition to conventional rehabilitation. A control group had conventional rehabilitation with gait training without RAS (acoustic cue). The posttest was conducted 1 day after the 6-week intervention period. All assessment data were collected by 3 physical therapists who were blinded to the treatment allocations. The training was conducted by 2 therapists, and the therapists were not blinded. This study was only assessor-blind. All assessments were performed by 3 physiotherapists who were blinded to training group assignments. Subjects who were unable to participate in the study or who were not tested after the training were excluded from the final analysis. In the GTBR group, the final analysis was performed on all 23 patients. In the control group, 21 patients were excluded because of discharge from the hospital (Figure 1).

### 2.3. Procedure Auditory Stimulation Sound Production Process

In order for us to determine walking speed, subjects walked at various speeds without an assistant or support, and the most comfortable speed for each subject was set. While patients were walking at a comfortable speed, the step times of both legs were calculated using a gait analysis system (OptoGait, Microgate S.r.l, Bozen, Italy). Based on the calculated step time, the auditory stimulation sound was produced at an increased rate of 10% for the paretic side and 5% for the non-paretic side, rather than at a comfortable speed. Previous studies have shown that gait velocity and symmetry improved when patients walked to a faster acoustic cue than at a comfortable speed. To reduce the asymmetry of both step times, fast acoustic cues of 10% and 5% were applied differently. The auditory stimulation sound was generated using digital audio editing software (GoldWave v5., GoldWave Inc., St. John’s, NL, Canada). We provided different pitch sounds to distinguish the sounds applied to both legs. Measurements were taken every 2 weeks and acoustic cues were given to subjects according to the changed step time.

### 2.4. Gait Training with Bilateral RAS (GTBR) 

GTBR was performed once for 30 min, and the training time consisted of warm-up for 5 min, gait training for 20 min and cool-down for 5 min. The warm-up was done to increase adaptability to RAS and to allow practice with the RAS beat. Another purpose of the warm-up was to reduce the spasticity of the legs. During the gait training, the auditory stimulation sound was set for each individual to be used. Before the gait training, the participants nodded their heads in a sitting position in accordance with the sound, tapping the floor with their feet in a sitting position, and marched in place for the adaptation of RAS.

The gait training was performed in an independent treatment space with an elliptical track structure, including a linear section of 3300 cm and a rotation section of 879.2 cm. While subjects walked, each sound was adjusted to the heel strike of each foot.

To prevent disturbance due to external interference and noise during walking, we used a Bluetooth wireless headphone (MDR-RF4000K, Sony, Tokyo, Japan) for the subjects to hear the sound. One physical therapist performed a heel strike on the patient’s bilateral RAS and confirmed that the subject walked well to the bilateral RAS via the same Bluetooth wireless headphone.

Before the experiment, subjects did not receive any training or electrical stimulation that could affect walking, and all subjects were asked to wear the same type of shoes for accurate measurement. If anyone felt vertigo during training or was tired and unable to walk any longer, they were given enough rest.

### 2.5. Conventional Rehabilitation

Conventional rehabilitation programs consisted of therapeutic exercise, occupational therapy, and electrical stimulation therapy. Therapeutic exercise was based on proprioceptive neuromuscular facilitation and consisted of upper extremity movement. Occupational therapy consisted of upper extremity functional exercise to improve activities of daily living. Electrical stimulation therapy consisted of applying a passive functional electrical stimulus to the wrist. Each exercise consisted of 30 min of therapeutic exercise, 20 min of occupational therapy, and 10 min of electrical stimulation therapy.

### 2.6. Outcome Measurements

To collect data for quantitative gait analysis, a gait analysis system (OptoGait) was used to measure temporal and spatial gait ability. OptoGait was used in this study and consisted of two 3-m-long transmission bars and a webcam (Logitech Webcam Pro 9000, Logitech International S.A., Lausanne, Switzerland). The width of both bars was 1 m. Each bar communicated via infrared sent to the reception bar from the transmission bar light-emitting diode (LED) set 1 cm apart. The gait of the patients was detected between the transmission bars that collected the data on time and spatial variables. The webcam saved the video information and accurately synced the gait (i.e., order of the feet and error recognition from overlapping feet). The collected data on time and spatial variables were processed by OptoGait Version 1.5.0.0. The software was also used to connect the long axis of the reflective markers attached to the lower extremities to measure the flexion angle of the knee joint in the stance phase.

Gait symmetry was calculated with the data collected by the gait analyzer software as described below. The symmetry index (SI) is the ratio of the sum and difference between the non-paretic and paretic side values [24]. GA was calculated by multiplying the log value of the paretic/non-paretic side value by 100 [25]. The symmetry ratio (SR) was obtained by dividing the paretic side value by the non-paretic side value. In this study, both sides’ step times were used in calculating the symmetry [26].

Symmetry Index (SI) = [(Paretic − Nonparetic)/(0.5(Paretic + Nonparetic))] × 100%
(1)

Gait Asymmetry (GA) = |100 × [ln(Paretic/Nonparetic)]|
(2)

Symmetry Ratio (SR) = Paretic/Nonparetic
(3)

To assess balance ability, we used the Timed Up and Go test (TUG) and Berg Balance Scale (BBS). The TUG was measured when the participant sat on a chair with armrests and, upon hearing the command “begin”, got up from the chair, walked to the 3-m mark ahead, returned to the chair, and sat down, with time measured from start to finish for each participant to do so.

The BBS is used to assess the balance of older people and people with neurological disorders who are at high risk of falling down. It is a functional balance test that considers 3 aspects: Maintenance of posture, postural control through voluntary exercise, and reaction to external stimuli. The BBS has 14 items: Movement from sitting to standing, standing unsupported, sitting with back unsupported, movement from standing to sitting, transfer from one chair to another, standing with eyes closed, standing unsupported with feet together, standing and reaching forward with outstretched arm, standing and picking up an object from the floor, turning to look behind over the left and right shoulders, turning 360°, alternating feet placed on a platform, standing with one foot in front, and standing on one leg. Each item is scored on a scale of 0 to 4, with a total score of 56 [27].

Lower extremity function was measured by the Fugl–Meyer Assessment (FMA). The FMA is used to assess motor recovery of the lower extremities, which comprises 7 items with respect to the hip, knee, and ankle: 3 with reflex, 3 with flexor synergy, 4 with extensor synergy, 4 with volitional movement, 1 with normal reflex activity, and 3 with coordination (maximum score 34 points) [28].

### 2.7. Statistical Analysis

All statistical analyses were performed using SPSS Version 19.0 (SPSS Inc., Chicago, IL, USA). The Shapiro–Wilk test was used to confirm the normal distribution of all outcome variables. The paired *t*-test was used to compare dependent variables within groups, whereas the independent *t*-test and chi-squared test were used to compare dependent variables between the 2 groups. Statistical significance was set at *p* value < 0.05.

## 3. Results

One of the 22 participants in the control group was excluded from the analysis because the patient was discharged from the hospital. Accordingly, a total of 44 participants were included for analysis, of whom 23 were in the GTBR group and 21 were in the control group. There were no significant differences in the general characteristics of both groups and dependent variables between the two groups (Table 1).

Outcome measures of gait symmetry, gait ability, balance ability, and lower extremity function of the GTBR and control groups are shown in Table 2.

Gait symmetry on step time was significantly improved in the GTBR group relative to baseline (*p* < 0.05). In addition, the magnitude of the decreases in gait symmetry on step time was significantly greater in the GTBR group relative to that in the control group (*p* < 0.05). However, gait symmetry on step length did not significantly improve in either the GTBR or the control group.

Gait ability on velocity and cadence was significantly improved in both groups relative to baseline values (*p* < 0.05). However, the GTBR group showed significantly greater improvement in comparison to the control group in velocity and cadence (*p* < 0.05).

Both groups showed significant improvement in the TUG test, BBS, and FMA relative to baseline values (*p* < 0.05). However, the difference in change between groups was not statistically significant.

## 4. Discussion

In comparison to standard gait training, a 6-week program including GTBR proved to be more effective for stroke patients’ gait symmetry, gait ability, balance ability, and lower extremity function.

GTBR, a way of inducing a synchronization between RAS and motor areas, promoted stroke patients’ gait symmetry. It also improved their gait ability, balance ability, and lower extremity function. Additionally, the control of gait pattern with use of bilateral RAS ameliorated the GA of stroke patients. It was demonstrated that its influence can be effective in rehabilitation programs for stroke patients.

The auditory cues used in previous studies utilized the cadence of the subjects in experiments, and they were measured by stopwatch or gait measurement sensors. Compared with previous studies [29,30,31], this study was applied according to the step time that was calculated by the gait analysis system. On the paretic side, RAS with a 10% faster tempo than step time was applied, and on the non-paretic side, RAS with a 5% faster tempo than step time was applied.

The present study also applied a faster tempo than the average tempo, which was suggested by existing studies on auditory stimulation. Because previous studies used various tempos and gait training was conducted on a treadmill [29,30,31], it was not possible to accurately suggest which tempo would be more effective in a case of normal gait. However, in consideration of the application results of this study, it could be suggested that the application of auditory stimulation with a faster tempo to the paretic and non-paretic sides would be an effective rehabilitation program for stroke patients.

In this study, the gait symmetry of step time in the GTBR group increased significantly, but that of step length showed no change. In order to investigate change in symmetry, this study utilized the changes in time and length values based on step. In this comparison, previous research generated a symmetry ration with the use of values of stance time, swing time, and stride length. Thus, it is impossible to directly compare existing research with the results of the present study. However, through GTBR, it could be confirmed that it would be possible to improve the asymmetric gait pattern of stroke patients.

Although symmetry of step time improved through GTBR, symmetry of step length saw no change. This was because auditory stimulation was created with the standard of step time. As gait training was performed in accordance with auditory stimulation with rapid speed, which was designed on the basis of step time of both paretic and non-paretic legs, the step time of the experimental group could be controlled. Subsequently, gait speed actually increased due to the gait performed at the controlled speed. This experimental results show that, because of auditory stimulation, the speed of step time increased, and at the same time, temporal symmetry of the gait pattern of stroke patients developed.

According to the study by Patterson et al. [24], people normally showed 1.03 points of a step length’s SR, but stroke patients revealed 1.13 points of a step length’s SR. The researchers reported that, if SRs of paretic and non-paretic sides were close to 1 point, it could be regarded as normal and symmetric. In this study, the experimental group represented a significant SR decrease from 1.14 to 1.11. With GTBR, stroke patients’ gait symmetry improved. Gait training, which was performed in accordance with auditory stimulation of a regular tempo and faster speed, affected pattern generation so as to change the gait pattern shown before the training. GTBR improved the asymmetric gait pattern of stroke patients and developed movement of the paretic side. In consideration of the results, it is regarded that gait symmetry improved.

There was no change in the symmetry of step length in this study. With this result, it was difficult to improve the gait symmetry of spatial parameters using only step time, the standard of auditory stimulation. It can be assumed that a certain training method based on spatial data, not a change in gait pattern using auditory stimulation on the basis of step time, that is, a kind of temporal data, is needed. The step time utilized in this research is a type of temporal data depending on the movement of the lower extremities during walking, so it is a method that can be insufficient in changing step length, that is, a kind of spatial index. In this regard, it can be said that research is required to achieve both spatial symmetry and a rehabilitation effect at the same time.

As a result of this study, both groups showed significant improvements in gait ability. This was demonstrated in the comparison between the two groups. Throughout the gait training, while doing heel strikes according to bilateral RAS of a faster tempo than a normal step time, patients moved their lower extremities faster than usual. As such rapid movement of lower extremities was repeated, gait patterns changed, and gait speed increased to the speed that was similar to the tempo of the given auditory stimulation. Moreover, with stepping in accordance with the auditory stimulation of a rapid tempo, stride length changed, and cadence increased.

In the study by Hurt et al. [32], the group who underwent gait training that applied RAS showed an increase in speed from 38.8 m/min to 57.6 m/min after the training. In the experiment by Schauer and Mauritz [19], speed increased by 27%. In addition, in the study by Thaut et al. [11], a group performing gait training with RAS showed a significant increase in gait speed, presenting a change from 14.1 to 34.5 m/min throughout the training. By contrast, the control group did not improve significantly. As suggested by the similar results of their 6-week research, Thaut et al. [15] asserted that the amount of the increase was higher, and the period of the training affected the results.

GTBR improved one leg stance, so that the change in gait pattern increased gait ability, such as stride length and stride time [22]. Then, paralysis of both upper and lower extremities decreased [33], and due to the increased control ability of the trunk and lower extremities, stride length and time were enhanced [20]. It can be thought that GTBR induced changes in gait pattern, and movements of the trunk and lower extremities were improved, and gait ability became better. With the change in gait pattern, which resulted from auditory stimulation, a direct improvement appeared in cadence. Furthermore, auditory stimulation of a rapid tempo improved gait speed.

To examine one’s balance ability, this study utilized TUG and BBS to prove that GTBR improved the balance ability of stroke patients. This improvement was also shown in the control group. Balance and gait abilities are significantly correlated, and both gait speed and balance ability increased [34]. The amounts of the increase in gait speed and balance ability showed a similar tendency. Our results were similar to the work of Combs et al. [35], which found that gait training improved BBS by 4.2 points and speed changed from 0.62 to 0.73 m/s. Thus, it can be observed that both gait and balance improved simultaneously. Next, in the research by Yavuzer et al. [36], movement of the pelvis was improved after the training, which aimed to enhance balance.

As movements of the upper and lower extremities as well as thoracic spine rotation with an axis of the pelvis were developed by GTBR, movement of the trunk enhanced so that balance ability finally improved. Additionally, pelvic paralysis decreased through GTBR. Subsequently, with stability and development in functions of the hip joint, compensation activity decreased, and balance increased. Increase in the activities for maintaining balance affected balance ability [33,37]. With gait training for 6 weeks, lower extremity function and trunk control ability developed. 

To examine how GTBR would change lower extremity function, this study used the FMA scale. In this study, both the GTBR and control groups showed significant improvements, and there was no difference between the groups. Noting the significant effects in both groups, it could be observed that repeated gait trainings were required for functional improvement of the lower extremities. Moreover, a greater change was found in the values calculated within the GTBR group, so it was confirmed that GTBR was an effective method for development of lower extremity function. If training were to be performed for a longer term than that in the present study, it could be suggested as an effective rehabilitation program for functional recovery of stroke patients’ lower extremities. 

Effects of GTBR from this experiment are not generalizable because our sample size of stroke patients was small. We suggest that the use of GTBR should be applied to a larger sample size and more intimately explore the effects of functional improvement similar to balance and lower extremity function.

## 5. Conclusions

GTBR is useful as a rehabilitation program for functional locomotor recovery of stroke patients. It can also be used as a home exercise program for outpatients. Future studies may follow four directions to further establish the role of RAS in gait rehabilitation: (1) To examine specifically the effects on symmetry, gait variables should be analyzed in various ways; (2) To discover the most effective tempo, a variety of auditory stimulation tempos should be applied; (3) A study providing visual cues for effects of step length should be conducted; (4) To explore the effects on balance and lower extremity functions, long-term training should be performed.

## Figures and Tables

**Table 1:** General characteristics of subjects.

	GTBR Group (*n* = 23)	Control Group (*n* = 21)	x^2^/t
Gender (male/female)	13/10	11/10	0.783
Age (years)	56.00 ± 9.39	54.92 ± 6.65	0.649
Height (cm)	165.30 ± 6.71	167.04 ± 3.82	0.279
Weight (kg)	65.65 ± 7.76	69.46 ± 7.35	0.091
Duration of stroke (month)	14.22 ± 5.79	14.29 ± 5.16	0.963
Paretic side (left/right)	15/8	13/8	0.820
Lesion site (MCA/ACA)	20/3	17/4	0.587
Lesion type (Infarct/hemorrhage)	19/4	18/3	0.778

Values are expressed as mean ± standard deviation (SD). The independent *t*-test and chi-squared test are compared dependent variables between group. GTBR = gait training with bilateral rhythmic auditory stimulation, MCA = middle cerebral artery, ACA = anterior cerebral artery.

**Table 2:** Comparison of secondary measures within group and between groups.

Variables	GTBR Group (*n* = 23)			Control Group (*n* = 21)		
	Baseline	Post	Change Score	Baseline	Post	Change Score
Gait symmetry on step time						
SI (%)	0.03 ± 0.03	0.02 ± 0.03	−0.01 ± 0.01 *^†^	0.02 ± 0.02	0.02 ± 0.01	0.00 ± 0.01
GA (score)	5.18 ± 5.77	4.07 ± 5.29	−1.11 ± 1.33 *^†^	3.53 ± 2.75	3.56 ± 1.73	0.03 ± 1.50
SR (ratio)	1.14 ± 0.14	1.11 ± 0.13	−0.03 ± 0.03 *^†^	1.09 ± 0.07	1.09 ± 0.04	0.00 ± 0.04
Gait symmetry on step length						
SI (%)	0.02 ± 0.02	0.01 ± 0.02	0.01 ± 0.01	0.02 ± 0.02	0.02 ± 0.02	0.00 ± 0.00
GA (score)	2.72 ± 3.92	2.29 ± 4.09	−0.43 ± 1.71	3.96 ± 3.03	3.83 ± 2.75	−0.14 ± 0.48
SR (ratio)	0.98 ± 0.11	0.99 ± 0.11	0.01 ± 0.04	0.98 ± 0.11	0.98 ± 0.10	0.00 ± 0.01
Gait Ability						
Velocity (m/m)	50.01 ± 12.49	52.15 ± 13.23	2.14 ± 2.02 *^†^	48.50 ± 10.20	48.88 ± 10.19	0.38 ± 0.85 *
Cadence (steps/min)	97.10 ± 22.97	99.32 ± 23.89	2.23 ± 2.43 *^†^	96.05 ± 16.31	97.03 ± 16.82	0.98 ± 1.32 *
Balance ability						
TUG (sec)	25.68 ± 2.96	25.21 ± 2.91	−0.47 ± 0.64 *	26.68 ± 4.08	26.38 ± 4.13	−0.30 ± 0.61 *
BBS (score)	42.57 ± 4.78	43.70 ± 5.00	1.13 ± 1.49 *	41.58 ± 5.21	42.08 ± 4.96	0.50 ± 0.83 *
Lower extremity function						
FMA (score)	13.89 ± 4.76	14.76 ± 5.08	0.87 ± 1.52 *	13.75 ± 2.88	14.08 ± 2.87	0.33 ± 0.56 *

Values are expressed as mean ± standard deviation. SI = Symmetry Index, GA = Gait Asymmetry, SR = Symmetry Ratio, TUG = Timed Up and Go Test, BBS = Berg Balance Scale, FMA = Fugl–Meyer Assessment. * means significant difference within group. ^†^ means significant difference between group.
